# Changing patterns in diagnostic strategies and the treatment of blunt injury to solid abdominal organs

**DOI:** 10.1186/1865-1380-4-47

**Published:** 2011-07-27

**Authors:** Cornelis H van der Vlies, Dominique C Olthof, Menno Gaakeer, Kees J Ponsen, Otto M van Delden, J Carel Goslings

**Affiliations:** 1 Department of Surgery, Maasstad Ziekenhuis, Rotterdam, The Netherlands; 2 Trauma Unit Dept. of Surgery, Academic Medical Center, Amsterdam, The Netherlands; 3 Dept. of Emergency Medicine, Medisch Spectrum Twente, Enschede, The Netherlands; 4 Trauma Unit Dept. of Surgery, Medisch Centrum Alkmaar, Alkmaar, The Netherlands; 5 Dept. of Radiology, Academic Medical Center, Amsterdam, The Netherlands

## Abstract

**Background:**

In recent years there has been increasing interest shown in the nonoperative management (NOM) of blunt traumatic injury. The growing use of NOM for blunt abdominal organ injury has been made possible because of the progress made in the quality and availability of the multidetector computed tomography (MDCT) scan and the development of minimally invasive intervention options such as angioembolization.

**Aim:**

The purpose of this review is to describe the changes that have been made over the past decades in the management of blunt trauma to the liver, spleen and kidney.

**Results:**

The management of blunt abdominal injury has changed considerably. Focused assessment with sonography for trauma (FAST) examination has replaced diagnostic peritoneal lavage as diagnostic modality in the primary survey. MDCT scanning with intravenous contrast is now the gold standard diagnostic modality in hemodynamically stable patients with intra-abdominal fluid detected with FAST. One of the current discussions in the literature is whether a whole body MDCT survey should be implemented in the primary survey.

**Conclusions**

The progress in imaging techniques has contributed to NOM being currently the treatment of choice for hemodynamically stable patients. Angioembolization can be used as an adjunct to NOM and has increased the success rate to 95%. However, to date many controversies exist about the optimum patient selection for NOM, the proper role of angioembolization in NOM, the best technique and material to use in angioembolization, and the right follow-up strategy of patients sustaining blunt abdominal injury. Conducting a well-designed prospective clinical trial or a Delphi study would be preferable.

## Introduction

Trauma is the leading cause of death among people who are younger than 45 years [1]. One of the main causes of death after trauma, with numbers ranging from 40 to 80%, is exsanguination caused by injuries to the abdominal organs.

The spleen and liver are the most commonly injured organs as a result of blunt trauma [2]. The kidney is also commonly injured [2].

Over the past 40 years, many changes in the primary survey and treatment of patients with blunt abdominal trauma have occurred. Traditionally, emergent laparotomy was the standard of care. Currently, nonoperative management (NOM) is the most common management strategy in hemodynamically stable patients. The aim of this review is to describe the shift in management of blunt abdominal trauma over the past decades and to discuss recommendations for the future. We have focused on the following abdominal organs: the liver, spleen and kidney.

## Results

### Primary care

Before the 1970s, the structure of the diagnosis and treatment of life-threatening injury was very dependent upon the physician. The turning point of this management style came with the introduction of the Advanced Trauma Life Support (ATLS) principles by Steiner and Collicott in 1978 [3]. With this ATLS protocol, a clear guideline for the optimal primary clinical survey of patients with life-threatening injury was developed. The goal of the primary survey is to quickly assess and stabilize the trauma patient. Structure, simplicity and a multidisciplinary methodology are essential to this approach. An important ATLS principle is: 'treat first what kills first.'

### Diagnostic strategies

Major changes in the diagnostics of hemodynamically stable patients with blunt trauma have occurred. Currently, the primary survey consists of a chest X-ray, X-rays of the cervical spine and pelvis, blood and urine samples, and a Focused assessment with sonography for trauma (FAST).

#### Diagnostic peritoneal lavage (DPL)

Formerly, diagnostic peritoneal lavage (DPL) was the procedure of choice for the quick diagnosis of a hemoperitoneum in patients with blunt abdominal trauma. DPL, first described in 1965, resulted in a decrease in mortality and morbidity following abdominal trauma [4]. In general, FAST examination has replaced the use of DPL, because DPL is an invasive procedure and provides no information about which organ is injured, resulting in a high rate of negative or non-therapeutic laparotomies [5].

#### FAST

FAST is useful in trauma evaluation to identify intra-abdominal fluid, a herald of significant organ injury, with a sensitivity of 90-93% [6,7]. FAST can be performed simultaneously with resuscitation efforts during the initial trauma management and can be completed rapidly. FAST is, therefore, also useful in hemodynamically unstable patients [8]. One of the strengths of FAST in this patient group is that it helps to direct the surgeon to the abdomen as a major source of blood loss when positive, thereby leading to early laparotomy rather than CT. Despite its efficacy and non-invasive character, FAST has several important disadvantages. First, FAST does not accurately detect the extent (grade) or the exact site of the organ injury. Hemoperitoneum detected with FAST in hemodynamically stable patients should be followed by a CT scan to evaluate the nature and extent of injury in more detail [9]. Second, its sensitivity for direct demonstration of blunt abdominal injury is relatively low (between 34% and 55%), since the presence of free fluid in sufficient quantity indirectly indicates intraperitoneal injury [10]. Other limitations of FAST include operator dependence, limited retroperitoneal accuracy, and poor scanning results in obese patients or patients with overlying wounds.

When the FAST is negative for hemoperitoneum, it is still debatable whether a computed tomography (CT) scan is required. Estimates for the presence of intra-abdominal injury in the absence of hemoperitoneum on FAST can be as high as 29% [11]. In a recent study, 13% of the patients with clinical signs of abdominal injury and a negative FAST for intra-abdominal fluid were shown to have significant injury upon CT scanning [12]. Therefore, hemodynamically stable patients with a negative FAST and a high clinical suspicion of splenic injury, for example, a seat belt sign or upper abdominal pain, should undergo routine CT scanning [13,14].

#### CEUS

An increase in the utilization of another radiological modality, the contrast-enhanced ultrasound (CEUS), could contribute to the shift towards NOM. CEUS is a real-life, non-invasive, bedside, radiation-free technique. Some studies suggest that CEUS is a good alternative to MDCT scanning for the evaluation of traumatic lesions in solid abdominal organs, especially in patients with contraindications for CT contrast agents and in hemodynamically compromised patients [15]. The exact place of CEUS in the diagnostics of patients with blunt abdominal injury should be further determined in the future.

#### Computed tomography

The introduction of helical tomography in the 1980s has improved the detection and classification of blunt abdominal injury [16]. Currently, multidetector computed tomography (MDCT) scanning with intravenous contrast is the gold standard diagnostic modality in hemodynamically stable patients with intra-abdominal fluid detected with FAST. MDCT scanning with intravenous contrast has numerous advantages. First, the detection of injuries related to the liver, spleen and kidney can be reliably determined, with a sensitivity of 90-100%. Second, active bleeding (a contrast blush), pseudoaneurysms and post-traumatic arteriovenous fistulas can be diagnosed, and the localization of these vascular injuries can also be established. Third, the MDCT scan plays a decisive part in the order of treatment if more than one injury is present [17].

Because of the technical developments that have resulted in a higher degree of resolution of the CT scan and in quicker scanning, the effectiveness of conventional radiology (X-rays and FAST) in the clinical ATLS approach has been challenged. One of the main reasons for this is the lack of any research that proves that the mortality and disability rates of injured patients decrease after the implementation of the ATLS concept [18]. One of the current discussions in the literature is whether a whole body MDCT survey should be implemented in the primary survey. Some authors recommend conducting a whole body MDCT (the so-called imaging survey) as the standard diagnostic tool during the early resuscitation phase for patients with polytrauma. They report that a MDCT scan of the chest or abdomen results in a change of treatment in up to 34% of patients with blunt trauma [19]. A 30% reduction in mortality using the whole body MDCT is also reported [20]. Other arguments in favor of an imaging survey are the reduction in time from admission to intervention and the possibility of managing hemodynamically unstable patients in the same way [21].

**Figure 1:**
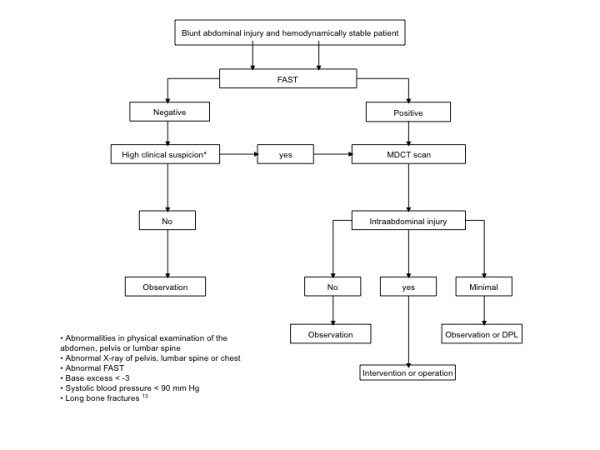
**Diagnostic algorithm of patients with blunt abdominal injury**.

It is debatable whether a whole body MDCT survey is to be recommended considering its disadvantages. The need for iodine-containing contrast and radiation exposure, especially in the relatively young trauma population, is not negligible when one considers the lifetime risk of cancer [22]. Moreover, whole body MDCT as part of the primary survey can only be adopted if an MDCT scan is available in, or very close to, the emergency department [23]. For the moment the benefit of whole-body MDCT scanning seems particularly high for patients with severe injury. The diagnostic algorithm for abdominal evaluation of hemodynamically stable patients after blunt trauma is depicted in Figure 1.

### Treatment

Historically, surgical management was the preferential treatment for most blunt abdominal injury, because NOM was associated with a high mortality rate [24]. However, many of the laparotomies were unnecessary and non-therapeutic [25]. With the wide availability and improved quality of CT scanning, and the more modern, less invasive intervention options, such as angioembolization, NOM has evolved into the treatment of choice for hemodynamically stable patients [26].

NOM consists of close observation of the patient completed with angioembolization, if necessary. Observational management involves admission to a unit and the monitoring of vital signs, with strict bed rest, frequent monitoring of hemoglobin concentration and serial abdominal examinations [27].

NOM, with or without angioembolization, is of benefit to trauma patients because the function in the organ concerned is preserved. In addition, the possible morbidity that may accompany a laparotomy, such as incisional hernia, abscess formation, pneumonia, wound infection, multiorgan failure, pancreatitis, bleeding, thromboembolic events and paralytic ileus, is avoided.

Angioembolization has proven to be a valuable adjunct to observational management and has increased the success rate of NOM to 95% [28]. The foundation for angioembolization was laid by Charles Theodore Dotter (1920-1985). In 1964 he performed the first transluminal angioplasty in a patient with peripheral occlusive disease [29]. Later on, the technique of embolization was introduced. The first application of embolization of the internal iliac artery in a patient with a pelvic fracture occurred in 1972, and from then on, the role of interventional radiology in the diagnosis and treatment of traumatic bleeding has increased significantly. Research demonstrates that angioembolization is a well-tolerated and effective tool in the treatment of traumatic liver, splenic and kidney injury [30-33].

**Figure 2:**
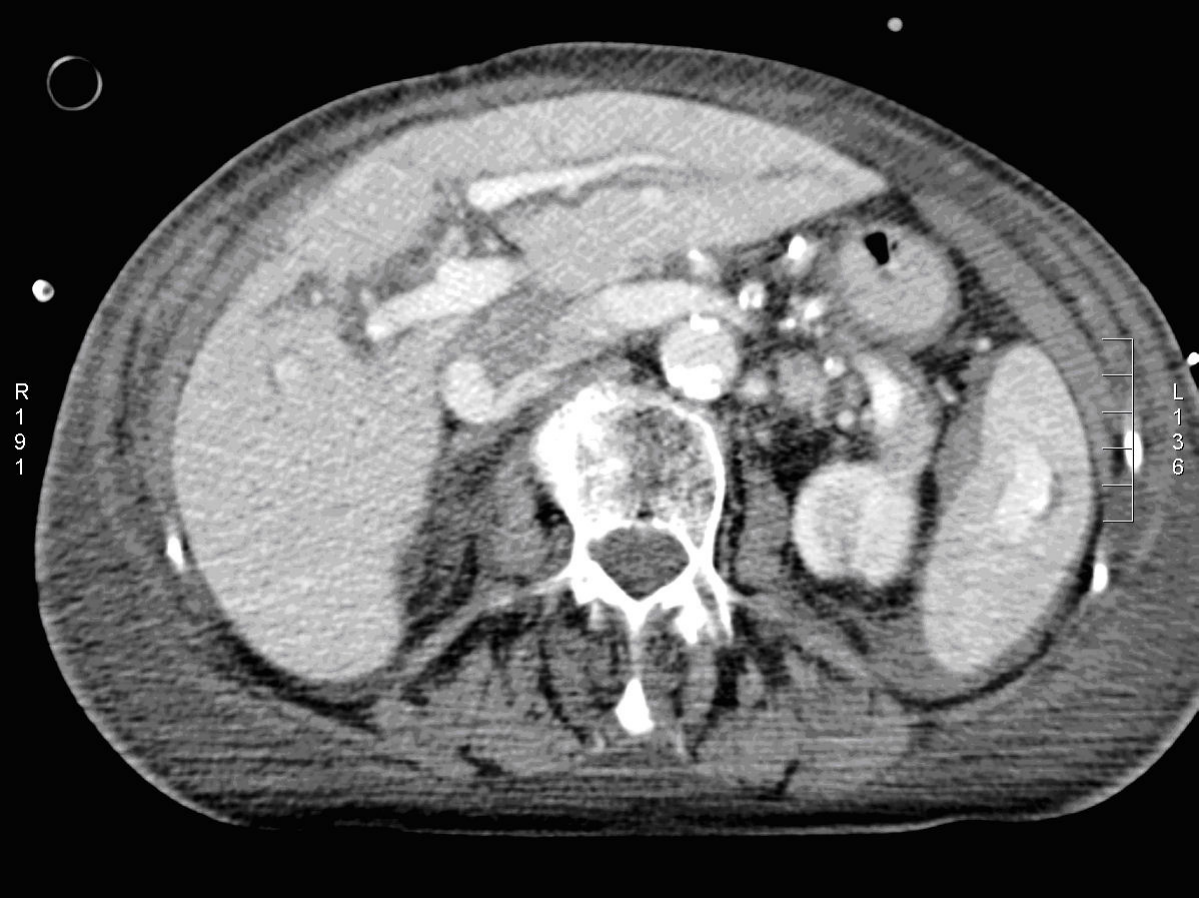
**Computed tomography with intravenous contrast shows small amounts of hemoperitoneum around the spleen and a contrast 'blush' confined to the splenic parenchyma**.

Determining which patients can benefit the most from angioembolization is still a controversial subject. CT features, such as a high grade of injury (AAST grade 3-5), pseudoaneurysm or arteriovenous fistula, contrast extravasation contained within the spleen (Figure 2), liver or kidney, and the presence of a hemoperitoneum, as well as patient characteristics such as age above 55 years old, GCS < 8 and male gender, are associated with an increased failure rate of NOM. Angioembolization can be advocated to improve the success rate of NOM in these patients [34-37].

**Figure 3:**
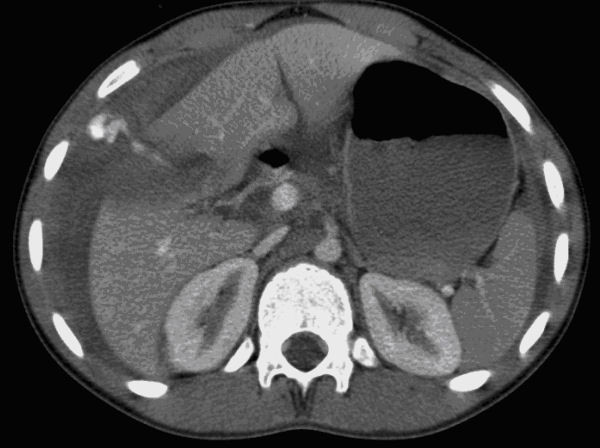
**Liver injury with intraperitoneal contrast extravasation visible on computed tomography scan**.

**Figure 4:**
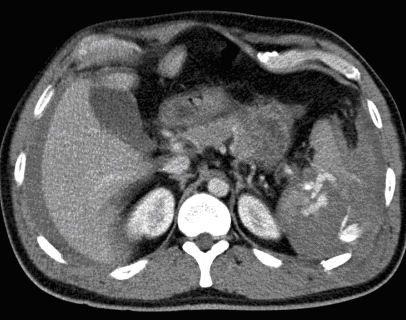
**Computed tomography with intravenous contrast showing hemoperitoneum, a fractured spleen with large hematoma and extravasation of contrast medium into the abdominal cavity**.

**Figure 5:**
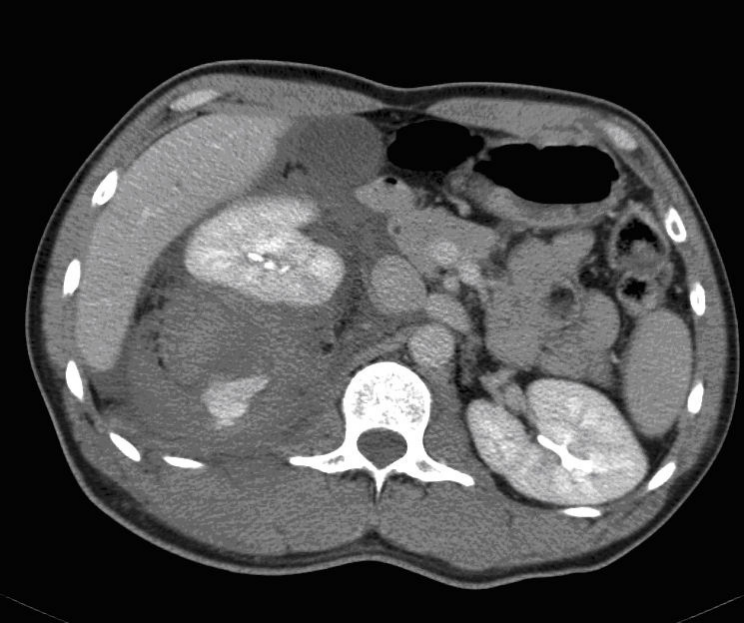
**Computed tomography with intravenous contrast demonstrating large hematoma around the right kidney with contrast extravasation**.

The single CT finding that warrants immediate angioembolization (or a laparotomy) is a contrast blush within the peritoneal cavity (Figures 3, 4, and 5).

#### Liver

The liver is frequently injured after blunt abdominal trauma [2]. Traditionally, a lesion of the liver was treated surgically. The major techniques that have been used over time are, in consecutive order, selective hepatic artery ligation and major liver resection using omental flaps for tamponade.

Ongoing bleeding, infections and the high mortality rate after operative treatment stimulated the search for alternative treatments, and, in 1990, NOM was introduced as a treatment for liver injury [38]. The high success rate (approximately 90%) combined with the lower mortality and complication rates, in comparison to surgical treatment, make NOM the treatment of choice for the majority of liver injuries, including high grade liver injury [39].

NOM consists of observation, supplemented by endoscopic retrograde cholangiopancreatography with the placement of a stent, or drainage by percutaneous transhepatic cholangiography if injury to the bile ducts has taken place. For active bleeds, angioembolization can be performed. Angioembolization may also be applied to control the hemorrhaging that may occur after damage-control operations using perihepatic packing in hemodynamically unstable patients.

Despite the reduction of mortality that has been achieved using angioembolization, some studies describe a rise in severe but treatable complications such as hepatic necrosis, abscesses or bile leakage [40-42]. Gallbladder ischemia, hepatic parenchymal necrosis and biloma may also occur, and in patients with a high grade liver injury (grade 4 and 5) the incidence of complications can be high [43].

#### Spleen

The spleen is the most frequently injured organ in blunt abdominal trauma, and a missed splenic injury is the most common cause of preventable death in trauma patients [44]. Formerly, in the early twentieth century, a splenectomy was nearly always performed. This invasive management was based on the following two findings: the first was the belief that the spleen could not heal spontaneously; the second was called the 'latent period of Baudet,' which refers to the tendency of the spleen to rupture at a later stage [45].

Changes to this type of management occurred in the 1970s when data about postsplenectomy complications were published describing the risk of overwhelming postsplenectomy infection (OPSI) and its high mortality rate [46]. In less than 10 years, NOM became the treatment of choice for splenic injury.

**Figure 6:**
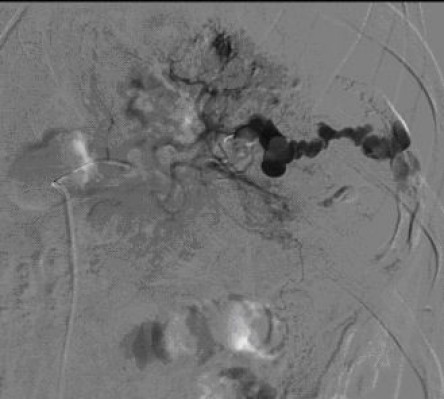
**Selective digital subtraction angiogram of the celiac axis showing the intra-peritoneal contrast 'blush' in the spleen, confirming active bleeding**.

**Figure 7:**
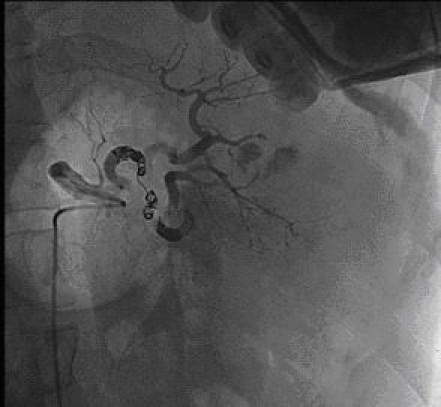
**Selective splenic angiogram immediately post proximal embolization demonstrating perfusion defects**. Contrast extravasation is no longer present.

In 1995, Sclafani described the first successful use of angioembolization in a patient with a splenic injury [47]. Since the 1990s, angioembolization has been frequently used to achieve better splenic salvages rates. To date, there is no consensus about the optimal localization of embolization, either proximal (Figures 6 and 7) or distal (selective), in the splenic artery.

A recent development is proximal splenic artery embolization (PSAE). The surgical equivalent of PSAE for splenic injury was first described in 1979 [48]. PSAE is predominantly used in cases with multiple disseminated hemorrhage sites or when quick intervention is needed because of the condition of the patient. Arguments in favor of proximal embolization are: the low failure rate, its speed, and the decreased incidence of splenic abscess or infarction [49,50]. PSAE does not significantly influence the splenic anatomy or the immune function in the long term [51]. A disadvantage of PSAE, however, could be that selective embolization in case of rebleeding is difficult, if not impossible, because the splenic artery cannot be accessed. Furthermore, ischemia of the pancreas (when embolization is performed proximally to the main pancreatic artery) and dislodgement of coils resulting in infarction of the spleen have been reported [52].

Selective embolization, used to stop focal bleeding, has also proved to be successful in NOM. This technique achieves hemostasis to the injured parts while preserving perfusion to the remainder of the spleen [53]. Disadvantages include the possibility of subsequent bleeding out of vascular injuries that were unnoticed owing to vasospasm [54] and the higher rate of minor complications such as infarctions [52]. However, the clinical relevance of these infarctions is questionable.

A recent meta-analysis showed that both techniques have an equivalent rate of major infarctions and infections requiring splenectomy [52]. However, the results regarding major rebleeding, the most common reason for failure of SAE [52], were inconclusive.

#### Kidney

The kidneys are affected in nearly 10% of all trauma patients, whereas blunt trauma is responsible for 90% of the renal injuries [55]. The switch from operative to nonoperative management for the treatment of renal injuries occurred as a result of critical perceptions. Researchers noticed that patients who underwent a laparotomy had a significantly higher risk of nephrectomy than the patients who were treated nonoperatively; it therefore seemed that maximal renal preservation, with a minimum of subsequent complications, could be better achieved with NOM [56].

In 2004, the Renal Trauma Committee and, in 2005, the European Association of Urology drew up guidelines for the optimum evaluation of patients with urological trauma [57,58]. The decisive factor in the evaluation is hemodynamic stability. Hemodynamic instability related to renal bleeding, complete ureteral tears or pelvic avulsions or leakage of urine into the peritoneal cavity are imperative indications for laparotomy. If the patient is hemodynamically stable, the distinction between gross or microscopic hematuria determines whether there is any further need for imaging and what the treatment options are. In case of gross hematuria, a MDCT scan is the gold standard for the evaluation of renal injury [58]. Microscopic hematuria does not demand imaging.

**Figure 8:**
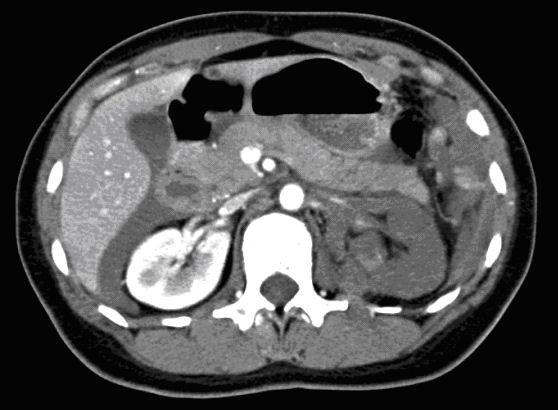
**Computed tomography with intravenous contrast: transection of the renal artery without contrast in the left kidney**.

**Figure 9:**
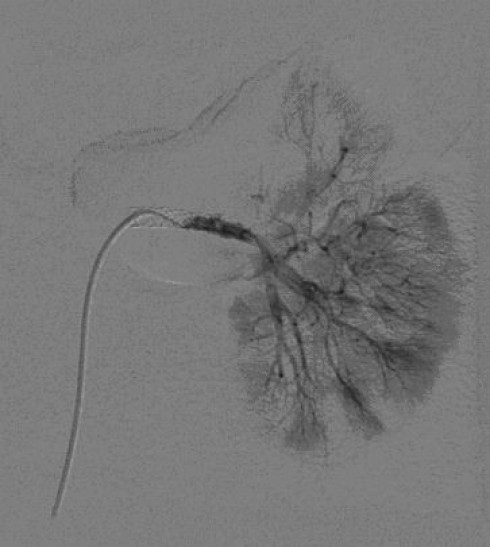
**Angiogram of the same patient as in Figure 5 after recanalization and placement of a stent in the renal artery, resulting in good perfusion of the kidney**.

Exclusion of coexisting injuries is of overriding importance in the initiation of NOM. Currently, NOM is used in up to 90% of renal injuries. This is because of the particularly high incidence of minor renal injury. Perinephric fluid collections or urinomas can be treated with percutaneous drainage. Patients with active hemorrhages detected on the MDCT scan can be treated with angioembolization of the renal arteries [33]. Kidney function can be preserved through recanalization and stenting even when a transection of the renal artery had been made (Figures 8 and 9).

## Discussion

Even though NOM has proven to be of tremendous benefit, a couple of controversies regarding the current management of trauma patients should be discussed.

Advances in CT technology have improved the practitioner's ability to determine the degree of injury and to identify patients who are more likely to fail NOM. However, until now, MDCT scanning has not been able to differentiate, in a precise manner, among which patients should be treated conservatively, which would benefit from angioembolization and which would respond best to a surgical response. The decision for treatment should always be based on the clinical situation and the physiological response of the patient to initial resuscitation.

A determinant of the success of NOM is the level of cooperation between different specialists in the hospital. Good teamwork among the trauma surgeon, the anesthesiologist and the (interventional) radiologist leads to a quicker understanding of the underlying injuries and thus shortens the time between entering the hospital and the initiation of therapeutic interventions. This seems obvious in level 1 trauma centers, but can be a matter of concern, especially in level II or II trauma centers.

### Recommendations for the future

The exact position of angioembolization in the NOM of blunt abdominal injury is still subject to discussion. Angioembolization has been shown to be a valuable adjunct to observational management and has increased the success rate of NOM in many series of clinical trials. However, a lot of controversies regarding angioembolization in patients with blunt abdominal trauma exist. Neither the optimal technique (proximal, distal or a combination of both) nor the material to use have been compared in a prospective trial with regard to outcome (success rate) and complication rate. A recently published systematic review and meta-analysis of Schnüriger et al. [52] is based on retrospective data, and the results regarding major bleeding, the most important reason for failure of SAE [52], were inconclusive.

The optimal follow-up strategy of patients sustaining blunt abdominal injuries has not been elucidated either. Up to now, the length of hospital stay, the need for, frequency of and best modality of follow-up imaging as well as discharge instructions with regard to resuming of activities are at the discretion of the physician. Research shows that practice patterns between physicians are quite variable [59].

Although difficult to conduct because of the nature of the trauma population, prospective (clinical) trials are necessary to determine the optimal patient selection for angiography and embolization, the most favorable technique and material to use for angioembolization, and the follow-up strategy in patients with traumatic blunt injury. One way of tackling this issue would be to conduct a Delphi study. The Delphi method is a systematic interactive forecasting method for obtaining experience-based agreement from a panel of independent experts. The process allows anonymous, non-biased consensus building and has been well validated for systematically assessing and organizing expert opinion [60]. Although low in level of evidence, we hold this study design appropriate since many of the controversies regarding the clinical decision making could be resolved by an international expert panel, selected on the basis of extensive clinical and/or research experience. We recommend a study such as this to be performed.

Furthermore, we advocate the improvement of logistic factors. If MDCT scans were present and available in trauma resuscitation rooms, the 'one hour rule' would be easier to fulfill. The MDCT scan could also play a part in the diagnostics of hemodynamically unstable patients [61]. At present, these patients go straight to the operating room; however, in the future they might also be treated with angioembolization.

## Conclusion

Over the past several years, major changes in the management of blunt abdominal injury have occurred. Because of the progress that has been made in the quality and wide availability of the MDCT scan combined with minimally invasive intervention options like angioembolization, NOM has evolved to be the treatment of choice for hemodynamically stable patients. NOM is a safe treatment for stable patients with traumatic liver, splenic or kidney injuries, and success rates of up to 95% are described in the literature. However, to date many controversies exist about the optimum patient selection for NOM, the proper role of angioembolization in NOM and the right follow-up strategy.

## List of abbreviations

NOM: nonoperative management; CT: computed tomography; ATLS: advanced trauma life support; FAST: focused assessment with sonography for trauma; DPL: diagnostic peritoneal lavage; CEUS: contrast enhanced ultrasonography; MDCT: multidetector computed tomography; OPSI: overwhelming postsplenectomy infection; PSAE: proximal splenic artery embolization

## Competing interests

The authors declare that they have no competing interests.

## Authors' contributions

CHV was responsible for the manuscript and carried out the writing process. DCO collected relevant articles, provided a great contribution to the writing process and took care of the word processing and layout. MG was involved in drafting the manuscript and created the reference list. KJP participated in the design of the study and gave valuable additions with respect to the content. OMD provided the figures and shared his expertise with regard to the diagnostics strategies. JCG conceived of the study, participated in the design of the study and revised it critically for important intellectual content. All authors read and approved the final manuscript.
